# The Involvement of Peroxiporins and Antioxidant Transcription Factors in Breast Cancer Therapy Resistance

**DOI:** 10.3390/cancers15245747

**Published:** 2023-12-08

**Authors:** Lidija Milković, Monika Mlinarić, Ivan Lučić, Ana Čipak Gašparović

**Affiliations:** Division of Molecular Medicine, Ruđer Bošković Institute, 10000 Zagreb, Croatia; lidija.milkovic@irb.hr (L.M.); monika.mlinaric@irb.hr (M.M.); ivan.lucic@irb.hr (I.L.)

**Keywords:** aquaporin, peroxiporin, NRF2, FOXO

## Abstract

**Simple Summary:**

The main problem preventing successful cancer treatment is therapy resistance. Initially, the membrane pumps were identified as the cause of resistance, but today we know that this is not so simple. Tumors can have intrinsic or acquired resistance to therapy. Both can be caused by several factors acting in parallel and leading to the resistant tumor phenotype. Here, we point to the new players that are elevated in malignant breast cancer and could, therefore, support changes that lead to therapy resistance.

**Abstract:**

Breast cancer is still the leading cause of death in women of all ages. The reason for this is therapy resistance, which leads to the progression of the disease and the formation of metastases. Multidrug resistance (MDR) is a multifactorial process that leads to therapy failure. MDR involves multiple processes and many signaling pathways that support each other, making it difficult to overcome once established. Here, we discuss cellular-oxidative-stress-modulating factors focusing on transcription factors NRF2, FOXO family, and peroxiporins, as well as their possible contribution to MDR. This is significant because oxidative stress is a consequence of radiotherapy, chemotherapy, and immunotherapy, and the activation of detoxification pathways could modulate the cellular response to therapy and could support MDR. These proteins are not directly responsible for MDR, but they support the survival of cancer cells under stress conditions.

## 1. Introduction

Despite great efforts in diagnosis and treatment, breast cancer ranks first among newly diagnosed cancers in women and is the leading cause of death in women of all ages [1]. The most common classification of breast cancer is based on the expression of hormone receptors (estrogen receptor (ER) and progesterone receptor (PR)) and human epidermal growth factor receptor 2 (HER2), grouping it into luminal A (positive for ER, PR, low Ki67), luminal B (positive for all, ER, PR, high Ki67), luminal B HER2-positive (positive for ER, PR, and HER2, high Ki67), HER2-enriched which is negative for ER and PR, and triple-negative breast cancer (TNBC) (negative for all, ER, PR, and HER2) [2]. Being a heterogeneous disease, the effectiveness of the treatment is highly dependent on the breast cancer subtype and whether the cancer is localized or has spread to other sites in the body. Not only intertumor heterogeneity but also intratumor heterogeneity contribute to diverse treatment responses [3]. The patient’s prognosis is worse if the cancer has progressed and metastasized, particularly due to the development of new metastases [4] which can occur if therapy fails. In addition, patients with TNBC within the specified subtypes mentioned generally have a poorer prognosis as there are only a few targeted therapy options available [5]. The ineffectiveness of a therapy is caused by intrinsic or acquired therapy resistance. Intrinsic resistance refers to the natural ability of some subpopulations of cancer cells to resist therapy, a characteristic present before the commencement of any treatment, while acquired resistance develops in a cancer that has initially responded to the therapy used. Diverse factors contribute to therapy resistance, such as genetic and/or epigenetic alterations in signaling pathways, changes in the number of membrane receptors and detoxification enzymes that affect drug accessibility, as well as modifications to a more permissive tumor microenvironment. Both ER-positive and HER2-positive breast cancers frequently experience relapse following their initially effective targeted therapies due to intrinsic and/or acquired resistance. Alterations in the ER, even shifting ER-positive tumors to ER-negative, and mutations in HER2, along with alterations in the PIK3CA/mTOR pathway, contribute to the ineffectiveness of tamoxifen and trastuzumab therapies, respectively [6,7]. While some subtype-specific strategies to overcome resistance in breast cancer exist, there is still a need for a better understanding of the underlying mechanisms and their interconnections [8]. Here, we will focus on cellular-oxidative-stress-modulating factors such as transcription factors NRF2, FOXO family, and peroxiporins, known as cellular hydrogen peroxide controllers.

**Figure 1:**
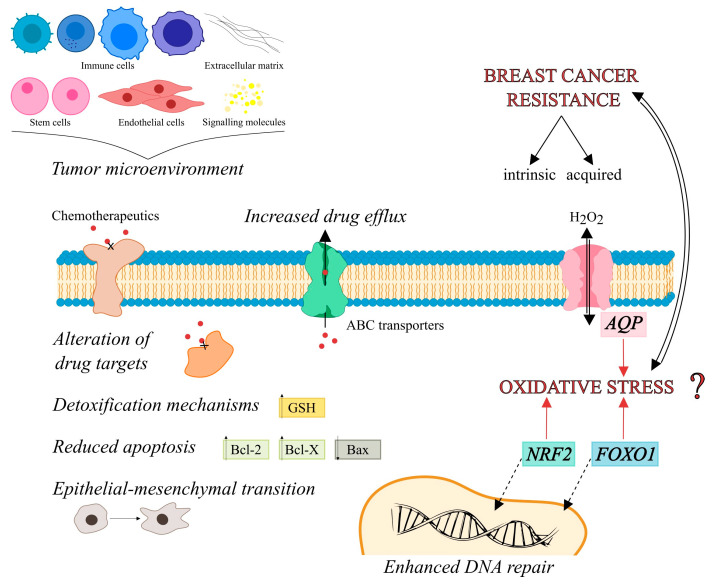
Multidrug resistance in cancer. Factors contributing to multidrug resistance are changes and interactions of tumor cells with cells in their microenvironment, changes in export pumps, enhanced detoxification systems, decreased apoptosis, epithelial–mesenchymal transition. Other factors modified due to oxidative stress that may increase resistance are peroxiporins (control of oxidative stress by channeling H_2_O_2_), antioxidant transcription factors NRF2 and the FOXO family.

Multidrug resistance is among the first mechanisms of therapy resistance to be described and is associated with Pgp pumps [9]. Since then, cancer resistance has been extensively studied and is now associated with complex changes that go beyond Pgp pumps, and are more complex than was originally thought (these changes are discussed in many review papers [10,11,12]). Here, we will present new pathways that may play a role in therapy resistance or support acquired resistance (Figure 1). One of the factors that modulates (cancer) cell pathways and responses is oxidative stress. Considering that cancer treatments are either based on oxidative stress (radiotherapy) or cause oxidative stress in addition to their original mechanism of action (chemotherapy and immunotherapy) [13,14], it is not surprising that factors that control and modulate oxidative stress may contribute to cancer resistance. The response to oxidative stress is universal regardless of the cell type, but the levels of specific components vary between cell types. Therefore, cancer cells may have increased certain components of the antioxidant defense system which may give them an advantage in surviving oxidative-stress fluctuations in the microenvironment (e.g., oxidative stress due to immune cell infiltration). In addition to increased components of antioxidative defense, cancer cells also have increased levels of transcription factors that regulate these components. NRF2 is the major antioxidant transcription factor, showing high expression in all types of breast cancer [15]. The FOXO family of transcription factors also respond to oxidative stress and control part of the antioxidant response. The regulation of the FOXO family activity is quite complex, and it is, therefore, difficult to determine the role and conditions in tumors under which they provide protection. In addition to transcription factors, enzymes and peptides, membrane pores can also regulate the flux of hydrogen peroxide across the membrane, and thus contribute to the control of oxidative stress. These pores are aquaporins with 13 isoforms in mammals (AQP0-AQP12), some of which have the ability to channel hydrogen peroxide and are called peroxiporins according to this ability.

## 2. Peroxiporins

Aquaporins (AQPs) were originally thought to be water channels, but as studies of aquaporins progressed, and new substrates were discovered, such as glycerol, these aquaporins were named aquaglyceroporins. In addition to the main substrate, all aquaporins can also channel other small polar molecules with different affinities [16]. Among the aquaglyceroporins, AQP3, AQP9, and especially AQP7 are primarily responsible for the permeation of glycerol. In breast cancer, the expression of these AQPs plays a crucial role in facilitating glycerol transport between cancer-associated adipocytes and cancer cells and supports metabolic reprogramming that promotes cancer cell proliferation [17]. Correlation-based network analysis, metabolomics, and gene expression data integration, identified key hubs, with AQP7 emerging as a regulator of external nutrients and cellular stress in breast cancer adaptation [18]. While AQP7 was not initially classified as a peroxiporin, a recent study indicates that AQP7 can act as one in bone marrow mesenchymal stem cells, playing a key role in their proliferation and adipogenic differentiation by regulating intracellular hydrogen peroxide levels [19]. Furthermore, exploiting its inhibition, given the contrasting effects observed in normal and malignant cells, could be particularly advantageous in combination therapy with mTOR inhibitors in tumors that are resistant to mTOR inhibitors [18]. Oxidative stress, an increase in ROS (Reactive Oxygen Species), contributes to the progression of breast cancer but also occurs during cancer therapy, exploiting higher states of oxidative stress in cancer cells to drive them to death. Hydrogen peroxide is the main ROS which facilitates signal transduction and thus influences cell fate decisions such as proliferation, migration, or cell death [20]. To harness the signaling effects of hydrogen peroxide, its formation must be controlled, including its flux in and out of cells [21]. Some of the aquaporins, called peroxiporins, facilitate the transport of hydrogen peroxide. Their ability to transport hydrogen peroxide is crucial for maintaining redox balance and contributes to the activation of signaling pathways that influence cell-fate decisions. Computer molecular dynamics simulations examining the permeation processes of hydrogen peroxide that occurs during cancer treatment with cold atmospheric plasma through AQP1 and the palmitoyl-oleoyl-phosphatidylcholine (POPC) phospholipid bilayer (PLB) suggest that the delivery should occur via AQP1 [22]. A combination of metadynamics and transition path sampling (TPS) methods revealed that hydrogen peroxide mimics the behavior of water for bidirectional transport through AQP3 [23]. Further research on mammalian cells has found that AQP8 and especially AQP3 are channels that facilitate the uptake of hydrogen peroxide, whereas this was not found for AQP1. Furthermore, the uptake of hydrogen peroxide mediated by AQP3 plays a role in regulating downstream intracellular signaling pathways, including the activation of AKT. However, even with the silencing of AQP3, the activation of AKT is not completely abolished, suggesting that other membrane transport mechanisms or aquaporins are involved in hydrogen peroxide transport [24]. Other studies confirmed this hypothesis, when it was shown that other AQPs such as AQP9 [25], AQP5, and AQP11 are involved in the transport of hydrogen peroxide across the membrane. In particular, AQP5 is involved in the response to oxidative stress and influences cancer cell adaptation and migration [26,27]. Unlike other peroxiporins, AQP11 is specifically found in the endoplasmic reticulum (ER) and partially accumulates in mitochondrial-associated ER membranes (MAM), where it transports hydrogen peroxide from ER to the cytosol. Its downregulation showed the disrupted movement of hydrogen peroxide through the ER but not through the mitochondrial or plasma membranes, suggesting that AQP11 specifically regulates redox homeostasis and signaling within the ER [28]. However, the recent research suggests that AQP11 regulates the transport of hydrogen peroxide from mitochondria to ER through an increased number of ER–mitochondria contact sites, which occurs independently of AQP11 expression [29].

In assessing the role of peroxiporins in breast cancer, it is, therefore, crucial to consider factors beyond mere expression. In addition to increasing their number, the cellular distribution of peroxiporins is also important. Current knowledge about peroxiporins in breast cancer is summarized in Table 1.

It is important to note that oxidative-stress-mediated anticancer therapy not only affects peroxiporins but can also affect the expression and function of other aquaporins. For example, brain metastases occur frequently in HER2+ breast cancer patients, leading to the frequent use of trastuzumab emtansine (T-DM1). Stereotactic radiosurgery (SRS) is usually used to treat these metastases. However, case series have reported increased toxicity when SRS is combined with T-DM1, and so caution is advised when combining these therapies. Mechanistically, T-DM1 targets reactive astrocytes and enhances radiation-induced cytotoxicity and astrocyte swelling by upregulating AQP4 [33]. These studies suggest that aquaporins, particularly peroxiporins, may modulate cancer cell fate and responses to therapy, and so we will provide an overview of each peroxiporin.

### 2.1. AQP1

Cytoplasmic expression of AQP1 is positively correlated with advanced pathological features of invasive ductal carcinoma (IDC) and indicates a worse prognosis for patients. In addition, lymph node metastases have higher cytoplasmic AQP1 expression compared to their primary tumors [34]. These events occur due to the ability of cytoplasmic AQP1 to recruit annexin A2 (ANXA2) from the cell membrane to the Golgi apparatus, resulting in Golgi apparatus extension. This process promotes migration and invasion of breast cancer cells. In addition, cytoplasmic AQP1 recruits cytosolic-free Rab1b to the Golgi apparatus, forming a ternary complex involving AQP1, ANXA2, and Rab1b. This complex facilitates the secretion of pro-metastatic proteins, including intercellular adhesion molecule 1 (ICAM1) and cathepsin S (CTSS). Cellular secretion of ICAM1 and CTSS further contributes to the migration and invasion of breast cancer cells [35]. AQP1-driven progression and spread of TNBC can be negatively regulated by receptor-interacting protein kinase 1 (RIPK1), a regulator of cell death. Binding of AQP1 to D324 of RIPK1 induces cleavage and inactivation of RIPK1, preventing the activation of RIPK1/RIPK3/MLKL-mediated necroptosis and RIPK1/caspase-8/caspase-3-mediated apoptosis. TNBC cells typically have high levels of AQP1 and low levels of RIPK1, which is associated with aggressive cancer features and poor prognosis. These findings shed light on the mechanism by which cytoplasmic AQP1 promotes TNBC progression and metastasis [36]. Knockdown of AQP1 leads to decreased proliferation and migration of MDA-MB-231 cells in vitro and attenuates tumor growth in mouse xenografts in vivo [37]. Furthermore, AQP1 is associated with estrogen-induced angiogenesis in ER+ breast cancer, as estrogen activates the estrogen-response element (ERE) in the AQP1 promoter and promotes AQP1 expression [38]. Furthermore, miR-320 negatively regulates AQP1 expression, and the downregulation of miR-320 and the upregulation of AQP1 are associated with worse prognosis in breast cancer patients [39]. Interestingly, AQP1 has also been suggested as a potential predictor of response to anthracycline therapy. In particular, patients with high AQP1 expression who received anthracycline therapy showed better clinical outcomes. AQP1 competes with glycogen synthase kinase-3β (GSK3β) for interaction, leading to the accumulation and translocation of β-catenin, thereby enhancing the activity of topoisomerase IIα (TopoIIα). This increases the sensitivity of breast cancer cells to anthracyclines. However, the expression of AQP1 can be inhibited by miR-320a-3p, resulting in reduced chemosensitivity [40].

### 2.2. AQP3

The expression patterns and subcellular localization of AQP3 are specific to different cell types and have been associated with cancer cell aggressiveness and mobility [41]. As a peroxiporin, AQP3 facilitates the transport of hydrogen peroxide and subsequently diverse signaling pathways. Similar to AQP1, estrogen promotes the expression of AQP3 by activating the estrogen-response element (ERE) in the AQP3 promoter. This upregulation of AQP3 affects the expression of molecules involved in epithelial–mesenchymal transition and actin cytoskeleton reorganization. Consequently, it enhances cell migration and invasion specifically in ER-positive breast cancer cells [42]. FGFR/PI3K and FGFR/ERK signaling pathways play a crucial roles in FGF-2-induced AQP3 expression and contribute to cell migration and metastasis in breast cancer [43]. AQP3-mediated modulation of the PI3K/AKT signaling pathway is specific to different breast cancer cell types [44]. Downregulation of AQP3 reduces proliferation, migration and invasiveness of the MDA-MB-231 TNBC cell line, and enhances the susceptibility of these cells to 5-FU [45]. In contrast, the cytotoxic effect of nucleoside-derived drugs, such as 5′-deoxy-5-fluorouridine (5′-DFUR) and gemcitabine, includes the induction of the expression of AQP3 in MCF7 cells, contributing to swelling and cell cycle arrest. This effect is not observed with cisplatin treatment [46]. TNBC is characterized by its aggressiveness and lack of hormone and HER2 receptors, making chemotherapy the primary treatment option. Due to the frequent recurrence and wide heterogeneity of TNBC, there is a need for new treatment strategies. A novel approach involves the use of bromodomain inhibitors, as bromodomains are involved in transcriptional regulation by binding to acetyl-lysine residues on histones. The combined effect of JQ1, a BET domain inhibitor, and GSK2801, a BAZ2A/B domain inhibitor, exhibits anti-proliferative activity in TNBC cells. Interestingly, this effect is associated with the upregulation of AQP3 in one of the TNBC cell lines tested, SUM159 [47]. The CXCL12/CXCR4 signaling pathway is also important for breast cancer cell migration and metastasis. AQP3 contributes to this process by transporting extracellular hydrogen peroxide produced upon CXCL12 activation of membrane NADPH oxidase 2 (NOX2). This leads to the oxidation of PTEN and protein tyrosine phosphatase 1B (PTP1B) and the subsequent activation of the AKT pathway. AQP3 knockdown decreased migratory ability and reduced the formation of spontaneous lung metastasis in orthotopic xenografts indicating the possible use of AQP3 as a therapeutic target [48]. Indeed, the use of the nanovehicle, which consists of different modules that ultimately regulate the release of the AQP3 inhibitor Auphen has shown reduced migratory ability in vitro and reduced lung metastasis in tumor-bearing mice [49]. In addition, AQP3 has shown to be a potential target for breast cancer stemness, as demonstrated by a study on the anti-cancer effects of cold atmospheric plasma (CAP). The AQP3/SCAF11/FOXO1 axis was identified as the mechanism by which CAP targets breast cancer stemness. CAP treatment suppressed AQP3-19Y phosphorylation, leading to the disruption of SCAF11-mediated AQP3-5K K48-ubiquitination and reduced FOXO1 stability. In addition, the CAP-generated reactive species entered cells via AQP3 and inhibited RPS6KA3, which is a shared kinase of AQP3 and FOXO1. Decreased AQP3-19Y phosphorylation impaired SCAF11-mediated AQP3-5K K48-ubiquitination, resulting in compromised FOXO1 activity in breast cancer stem cell maintenance. Indeed, CAP has been recognized as a stem-cell-specific treatment option. In combination with atorvastatin, which downregulates the PTEN/AKT pathway by promoting RhoB, CAP exhibited enhanced anti-cancer efficacy in both in vitro and in vivo settings [50].

### 2.3. AQP5

AQP5 is not only a water channel, but also regulates cell–cell adhesion proteins such as ZO-1, plakoglobin, β-catenin, and desmoglein-2, and overexpression of AQP5 showed that AQP5 interacts with them and contributes to their decrease [51]. It also regulates the polarity of breast cancer cells by interacting with and negatively regulating protein Scribble. Overexpression of AQP5 resulted in the decreased size and circularity of MCF7 spheroids, their RAS-mediated signal detachment and propagation, and decreased levels of Scribble [52]. Knockdown or mislocalization of Scribble from cell–cell junction promotes carcinogenesis of breast cancer carcinogenesis [53]. In breast cancer patients, high expression of AQP5 is associated with less-favorable survival rates. There is a positive correlation between high AQP5 expression and HER2 positivity, as well as AQP5 gene amplification suggesting AQP5 might be an oncogenic driver [54]. MCF7/ADR cells resistant to Adriamycin have higher expression of AQP5 than MCF7/S-sensitive cells. Silencing of AQP5 decreased proliferation, migratory and invasion ability, and induced apoptosis in MCF7/ADR suggesting that AQP5 is important for drug resistance, while its inhibition may enhance the chemosensitivity [55]. Adriamycin increases oxidative-stress parameters, which is the main reason for its cardiotoxicity [56]. One of the possible mechanisms by which AQP5 contributes to adriamycin could be the export of H_2_O_2_, thereby supporting antioxidant defense of tumor cell.

By targeting AQP5 expression, both miR-1226-3p and miR-19b-3p were found to decrease AQP5 levels and impede cell migration. Pathway enrichment analyses again showed that these miRNAs primarily regulate genes associated with the gap junction pathway. In order to efficiently deliver miRNAs targeting AQP5 to breast cancer cells, the researchers designed exosomes that express both miRNAs and a peptide targeting the interleukin-4 receptor, which is known to be highly expressed in breast cancer cells. These engineered exosomes successfully demonstrated an inhibitory effect on AQP5 protein expression and cell migration in MDA-MB-231 cells. Overall, this study highlights the potential of AQP5-regulating miRNAs as therapeutic agents to inhibit breast cancer cell migration, and exosome-mediated delivery as a promising approach [57].

### 2.4. AQP9

AQP9 is expressed in the digestive, nervous, and reproductive systems [58]. The expression of AQP9 in mammalian liver cells can be downregulated by insulin [59]. As aquaglyceroporin, AQP9 is also involved in the metabolism of glycerol in the liver [8]. However, the expression of AQP9 is downregulated in hepatocellular carcinoma (HCC) and the 5-year survival rate was significantly lower in patients with HCC and lower AQP9 expression than in patients with higher AQP9 expression. On the other hand, overexpression of AQP9 inhibited EMT, invasion and proliferation of HCC cell lines, while at the same time suppressing the Wnt/β-catenin signaling pathway which is often associated with tumor progression [58]. In HCC cells, lung carcinoma cells and leukemia cells, AQP9 was shown to be a transporter important for arsenic uptake, and its expression influenced the sensitivity of cells to arsenic treatment, thereby reducing the arsenic resistance of cancer cells [59,60,61]. This is consistent with evidence showing that AQP9 expression correlates with arsen trioxide (ATO)-mediated apoptosis in two cell lines of promyelocytic leukemia NB4 and HT93A [62]. Upregulation of AQP9 is also associated with increased sensitivity of colorectal cancer cells to treatment with 5-florouracil (5-FU) through increased uptake of 5-FU [63]. AQP9 is also considered to be a potential predictive marker for response to chemotherapy, as non-responsive patients with stage III colorectal cancer also had lower AQP9 gene expression [64]. ATO together with all trans-retinoic acid (ATRA) has been shown to block several cancer-associated signaling pathways and eliminate tumor-initiating cells in TNBC by targeting peptidyl-prolyl isomerase (PIN1), an enzyme involved in various cellular processes that are aberrant in tumor cells [65]. ATO can bind directly to Pin1 and, thus, inactivate its function. If the direct binding of ATO to Pin1 is disrupted, chemoresistance occurs. However, when TNBC cells are treated with ATO and ATRA, ATRA induces AQP9 and increases the uptake of ATO by cancer cells, while at the same time ATRA cooperatively inhibits Pin1 as well as other oncogenic pathways, such as NF-κB, β-catenin, c-Myc, Akt, and c-Jun [65]. Overexpression of AQP9 in TNBC has been shown to convert ATO-resistant cells into ATO-sensitive cells, while silencing of AQP9 leads to the inhibition of Pin1 degradation by ATO in ATO-sensitive cells [65]. Since there are results suggesting that AQP7 together with AQP9 may be a transporter of arsenite compounds into mammalian cells [66], this suggests there is a potential role of AQP9 and AQP7 in chemotherapy resistance.

### 2.5. AQP11 & AQP8

Studies on aquaporins focus on plasma membrane aquaporins, and there is not much data on intracellular ones. AQP11, one of the two unorthodox aquaporins, is located on the ER membrane, and is also a peroxiporin that regulates the flux of hydrogen peroxide across the ER membrane [16]. There are only a few studies that indicate a possible role of AQP11 in cancer. In lung cancer cell lines, AQP11 expression was positively correlated with cisplatin resistance and may be a predictor of cisplatin resistance in lung cancer [67]. In colorectal cancer, downregulation of AQP11 is associated with cancer progression acting through an axis along with miR-152-3p [68]. However, in breast cancer cell lines the expression of AQP11 was upregulated compared to the non-tumorigenic breast epithelial cell line [44] indicating the need to investigate this aquaporin. A study of Chetry et al. based on a database search indicated that high expression of AQP11, AQP8, AQP3, AQP5, AQP6, and AQP10 was correlated with better overall survival [69]. Mutations of AQP8, a peroxiporin found in mammals, have shown their influence on the mechanism of oxidative-stress-induced cell death in cancer cells treated with chemotherapy and radiotherapy. HeLa cells with an AQP8 mutation (AQP8 C53S) treated with X-radiation or ATO are more resistant to both treatments, suggesting that AQP8 permeability is a mechanism for protection against oxidative-stress-induced cell death after chemotherapy and radiotherapy [70].

Undoubtedly, peroxiporins contribute to the regulation of cellular oxidative capacity by regulating H_2_O_2_ flux through membranes and cell compartments. There is evidence suggesting that they also contribute to cancer development and progression, and that an increase in their expression may contribute to cancer resistance, but the exact mechanisms of action and their regulation are still far from understood.

## 3. Transcription Factors

**Figure 2:**
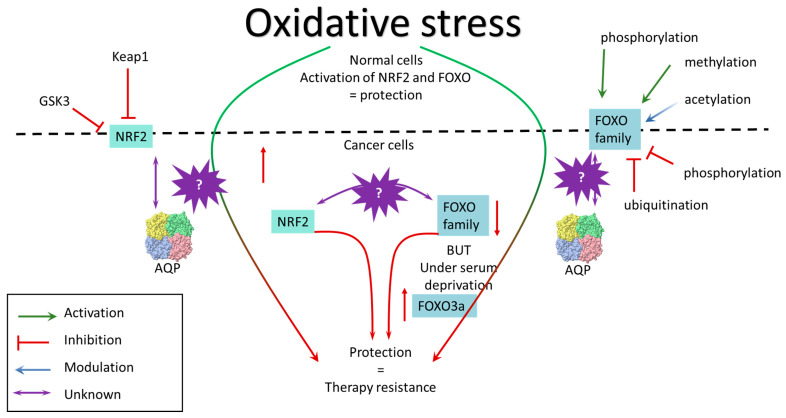
Antioxidant transcription factors in cancer cells. NRF2 is inhibited when coupled to KEAP1; in the nucleus, GSK3β phosphorylates NRF2 and inhibits it. Oxidative stress abolishes inhibition of Keap1 on NRF2 and activates it. Activation of NRF2 supports resistance to cancer therapy. On the other hand, FOXO regulation is more complex. Phosphorylation of certain sites can activate FOXO, while phosphorylation of other sites inhibits it. Methylation activates FOXO proteins, while ubiquitination degrades them. Acetylation modulates FOXO activity. Increased activation of NRF2 is observed in breast cancer and is associated with malignancy. The FOXO family is more complex as they were originally thought to be tumor suppressors, but there are situations where FOXO members are activated in tumors. There is no information on how these factors respond to the same (oxidative) stimuli and in the same system, and how they change in parallel, and how they regulate expression of aquaporins.

Antioxidant transcription factors act on electrophilic stimuli and activate the transcription of them in response to oxidative stress. When it comes to antioxidant transcription factors, NRF2 is considered the master antioxidant transcription factor, but the FOXO family also contributes to the neutralization of oxidative stress. These two transcription factors can respond to the same (oxidative) stimuli, but there are not yet many studies on their parallel effects and possible interactions [71]. Here, we will provide an overview of each of these transcription factors and their role in therapy resistance in breast cancer and, where possible, link them to peroxiporins (summarized in Figure 2).

### 3.1. NRF2

Nuclear Factor Erythroid 2-Related Factor 2 (NRF2) is a transcription factor that plays a critical role in cellular defense against oxidative stress by regulating the expression of various detoxification and antioxidant genes [72]. Its activity is tightly regulated to maintain a balance between normal physiological functions and the cellular response to reactive oxygen species (ROS). Under basal conditions, NRF2 is predominantly located in the cytoplasm, where it is bound to the Kelch-like ECH-associated protein 1 (KEAP1). KEAP1 acts as a substrate adaptor for cullin 3-based E3 ubiquitin ligase and facilitates the ubiquitination and subsequent proteasomal degradation of NRF2, ensuring its constant turnover and low abundance in the absence of ROS [73,74,75]. In the state of oxidative stress, KEAP1 acts as a redox sensor and undergoes conformational changes that prevent NRF2 binding, thereby pausing NRF2 degradation [73,76]. Consequently, NRF2 is stabilized and translocates to the nucleus, where it forms a heterodimer with small Maf proteins (sMaf) [72]. This complex then binds to antioxidant response elements (AREs) [77] in the promoter regions of target genes, such as NAD(P)H quinone oxidoreductase 1 (NQO1), heme oxygenase-1 (HO-1), and glutathione S-transferase (GST) [78,79] and initiates their transcription. In addition to KEAP1-mediated ubiquitination and degradation, there are other regulatory mechanisms. For example, the Bach family of transcription factors, including Bach1 and Bach2, compete with NRF2 for binding to AREs and they restrict the transcriptional activity of NRF2 [80]. Glycogen synthase kinase 3β (GSK3β) phosphorylates NRF2 within the nucleus, restricts its activity by degrading via the β-transducin repeat-containing protein-dependent pathway [81]. In addition, epigenetic modifications can alter the accessibility of AREs, thereby affecting NRF2 activity and the expression of its target genes [82]. Tight regulation of NRF2 activity is essential for the maintenance of cellular redox homeostasis. The repression of NRF2 ensures that its presence is low under basal conditions, while stress-induced changes allow NRF2 to activate cytoprotective and antioxidant genes. Several studies have reported a protective role of NRF2, with increased carcinogenesis under NRF2 deficiency [83,84,85].

Activation of NRF2 has been shown to protect healthy cells from carcinogens, which is why different activators are being investigated [80,86]. However, in pathological conditions, such as cancer, NRF2 activity is often dysregulated [87,88,89,90,91,92], and further activation could lead to the promotion of carcinogenesis [84,85,93]. Since NRF2 protects both healthy and cancer cells, it plays a dual role by acting as both a tumor suppressor and an oncogene [91,94]. The aberrant activation of NRF2 has been linked to cancer cell proliferation, metastasis, and increased resistance to therapy in various cancers. NRF2 exerts its protective effects by promoting the expression of genes involved in drug efflux transporters, detoxification enzymes, and antioxidant defense mechanisms [95,96,97,98,99,100,101]. Breast cancer resistance to therapies, including chemotherapy, radiotherapy, and immunotherapy, is a major challenge in the treatment of the disease, and understanding the role of NRF2 could provide valuable insights. Studies have shown that basal levels of NRF2 in different breast cancer cell lines correlate with their sensitivity to common cytotoxic chemotherapies [102]. This upregulation of NRF2 in cancer cells helps them to counteract the cytotoxic effects of therapies, especially those based on the generation of ROS (e.g., radiotherapy, chemotherapy, cisplatin and doxorubicin, tamoxifen), ultimately leading to reduced treatment efficacy [103]. Overexpression of NRF2 has been identified as a poor prognostic factor for solid malignancies, including breast cancer [104]. Tumors that overexpress NRF2 tend to have a worse clinical outcome, suggesting that NRF2 expression may serve as a prognostic marker for breast cancer patients [105]. Previously, we had found high NRF2 activation in all breast cancer immunophenotypes, and low NRF2 activation in the cancer stroma. However, the stroma of HER2+ and TNBC showed higher NRF2 activation than the stroma of hormone-receptor-positive breast cancer [15]. Numerous studies have demonstrated the role of NRF2 in mediating resistance to various chemotherapeutic agents. When NRF2 degradation is inhibited, a wide array of antioxidant genes such as *NQO1, thioredoxin*, and the *ATP Binding Cassette Subfamily C Member 1 (ABCC1)* drug transporter are upregulated, resulting in the creation of cells resistant to oxidative stress, benzo(a)pyrene, doxorubicin, and paclitaxel [106]. NRF2 expression was increased and associated with a resistant phenotype after prolonged incubation with doxorubicin and mitoxantrone [107]. Moreover, NRF2 expression is higher in doxorubicin-resistant cells than in doxorubicin-sensitive MCF-7 cells [108]. One study showed that stable overexpression of NRF2 in breast cancer cells resulted in increased resistance to cisplatin, doxorubicin, and etoposide. Conversely, downregulation of NRF2 either by overexpression of KEAP1 or by NRF2-specific small interfering RNA (siRNA), abrogated this resistance [101]. Another study confirmed these observations by showing that a reduction in NRF2 expression led to the reversal of doxorubicin resistance [109]. In addition to resistance, NRF2 silencing also suppresses colony/sphere formation and cell migration in vitro and tumor growth in vivo [110]. Furthermore, treatment with tert-butylhydroquinone (tBHQ) was found to increase the expression of NRF2 as well as its target genes, HO-1 and NQO-1. This NRF2 activation led to increased resistance of MCF-7 cells to doxorubicin [101]. Parthenolide was studied for the same purpose and exerted its anticancer activity by preventing NRF2 overexpression and promoting intercellular ROS accumulation [107]. Epigallocatechin-3-gallate (EGCG), a polyphenol found in green tea, increased NRF2 levels in MCF-7 and MDA-MB-231 breast cancer cell lines, leading to resistance to paclitaxel and doxorubicin in both cases [102]. Moreover, NRF2 activation in response to hypoxia-induced ROS accumulation confers resistance to cisplatin in breast cancer cells [111]. These results highlight the importance of NRF2 in mediating chemoresistance in breast cancer. Consequently, inhibitors targeting NRF2 could potentially serve as adjuvants in cancer therapy to increase the effectiveness of chemotherapeutic agents. By inhibiting NRF2, it may be possible to overcome resistance mechanisms and improve treatment outcomes. In addition, the involvement of NRF2 in immunotherapy resistance has been observed, highlighting its role in various resistance mechanisms. Breast cancer cells respond to tamoxifen-induced oxidation by increasing NRF2 expression and activating ARE. It has been shown that the expression of NRF2 target genes, such as *HO-1*, *thioredoxin*, and *peroxiredoxin*, is higher in tamoxifen-resistant MCF-7 cells than in control MCF-7 cells [112]. NRF2-initiated transcription of antioxidant genes and multidrug resistance transporters enables breast cancer cells to destroy or export toxic oxidation products [113]. As a result, these cells exhibit increased survival to tamoxifen-induced oxidative damage, contributing to tamoxifen resistance. Targeting NRF2 has shown promising results in reversing therapy. For example, a combination of NRF2 siRNA and EGCG downregulates intracellular NRF2 protein levels and reverses tamoxifen resistance in tamoxifen-resistant MCF-7 cells [114]. Another NRF2 inhibitor, brusatol, increased the anticancer activity of trastuzumab by inhibiting NRF2 [115]. NRF2 can mediate the response to radiotherapy. Following irradiation, expression of NRF2 and its target genes, *HO-1* and *NQO1*, was increased, which contributed to the elimination of ROS. These effects were reduced when NRF2 activity was suppressed by knockdown [116]. While its activation in healthy cells is crucial for maintaining redox homeostasis, dysregulation of NRF2 in cancer cells leads to therapy resistance via several mechanisms, including the activation of antioxidant pathways and multidrug resistance transporters. Strategies aimed at inhibiting NRF2 signaling have shown promise in suppressing cancer cell proliferation and sensitizing cancer cells to therapy [93]. Inhibition of NRF2 can be achieved using several approaches, including targeting upstream kinases, or directly blocking the NRF2-KEAP1 interaction [117]. Suppression of NRF2 activity compromises the resistance mechanisms employed by cancer cells and makes them more susceptible to the cytotoxic effects of therapeutic agents. Understanding the mechanisms by which NRF2 contributes to resistance and developing strategies to modulate its activity may prove promising in addressing the challenges of treating cancer patients and improving their outcomes.

### 3.2. FOXO

Forkhead box O proteins (FOXO) are a family of transcription factors, four of which are found in mammals (FOXO1, FOXO3, FOXO4, and FOXO6) [118]. These proteins have a highly conserved FH domain, which is a DNA-binding domain. The FH domain is located between the flanking N- and C- termini, and there are three other conserved regions, CR1, CR2, and CR3, in these two termini [119]. In addition, FOXO has a conserved nuclear localization sequence (NLS) and a nuclear export sequence (NES) [120]. The conserved regions, CR1 and CR2, harbor phosphorylation domains that are some of the most important regulation mechanisms [121]. Phosphorylation of these two domains by Akt/PKB enhances binding to 14-3-3 and its exclusion from and prevention from reentering the nucleus [120]. However, regulation of the FOXO family is far from simple and straightforward. FOXO proteins can be phosphorylated, methylated, acetylated, and ubiquitinated, and are regulated using different mRNA [119,122]. The complexity of FOXO regulation is reflected in the recent finding that the CR3 region of FOXO4 can bind to its FH domain, preventing it binding to DNA [123]. This autoregulation is prevented under oxidative stress when β-catenin binds to FOXO and promotes its activity [124]. The activity of FOXO under oxidative stress is important because FOXO targets antioxidant genes; both superoxide dismutases, *SOD1* and *SOD2*; peroxiredoxins, *Prx3* and *Prx5*; catalase, *CAT*; and glutathione peroxidase, *GPX1* [125]. Furthermore, oxidative-stress-based chemotherapy, such as doxorubicin, activates and translocates FOXO3a in the MCF7 breast cancer cell line [126]. However, it should be noted that the above mentioned post-translational modifications can either activate, inhibit, or augment FOXO activity, which is why the regulation of FOXO is referred to as the FOXO Code [119].

The FOXO code explains the different, sometimes antagonistic, activities of FOXOs [119]. FOXO proteins regulate cell cycle arrest and apoptosis, but also play a crucial role in differentiation [127]. FOXO homologues in *C. elegans* and the silkworm are active in lifespan extension [128,129]. In humans, FOXO3 is responsible for longevity [130]. In addition, FOXO can modulate cellular metabolism through post-translational N-acetyl-glycosylation, which affects the transcription of genes associated with energy metabolism [131,132]. Given these functions, the role of FOXO in cancer is not surprising. Initially, research on C. elegans and its FOXO ortholog DAF-16 indicated that FOXO regulates uncontrolled cell proliferation [133] suggesting that FOXO is a tumor suppressor. These conclusions were based on the high homology between DAF-16 and mammalian FOXO proteins, but once we have begun to uncover the complexity of FOXO interactions and discover the FOXO code, this view will be revised. Nevertheless, research on the role of FOXO in cancer is still inconclusive. For example, FOXO1 is associated with the “normal cell” phenotype in the liver, as its level decreases with the migratory potential of the hepatocellular carcinoma [134,135]. Furthermore, overexpression of FOXO1 in the hepatocellular cell line Hepa1-6 decreased their migratory potential when injected into mice [135]. FOXO1 can also mediate the induction of tristetraprolin (TTP), an mRNA-destabilizing factor that downregulates cancer-associated genes [136]. TTP responds to dexamethasone in MDA-MB-231 cells and reduces proliferation, and this effect is suppressed by the FOXO1 binding to its promoter, but only in the presence of dexamethasone [136]. In addition, miRNAs that are dysregulated in TNBC also target FOXO pathways [137,138]. In particular, miRNA-155 targets and downregulates the mRNA FOXO3a, among others, in chemo-resistant breast cancer cells [139]. These findings are confirmed by miRNA feedback inhibition of FOXO3a in the Heceptin-resistant HER2-positive breast cancer cell sublines SKBR3-pool2 (pool2) and BT474-HR20 (HR20) derived from SKBR3 and BT474 [140]. In addition to FOXO3a, FOXO1 was also downregulated by miR-96-5p in breast cancer, suggesting that regulation of FOXO proteins by miRNA plays an important role in tumor invasiveness [141,142]. However, studies suggest that the mutually antagonizing players FOXOs and c-Myc can work together under the control of epigenetic factors, to form an MLL2/FOXO/c-Myc axis that is activated by lapatinib, a drug used to treat HER2+ breast cancer, thereby reducing sensitivity to the drug [143]. In addition, numerous studies have shown that FOXO proteins act as tumor suppressors, but also that they can promote tumor growth and metastasis under certain conditions. Under serum deprivation, FOXO3a supports the induction of MMP9 and MMP13 and thus increases tumor invasiveness [144]. In addition to FOXO3a, FOXO1 is also involved in the regulation of matrix metalloproteinase. The study has demonstrated the association of Cdc25A, a cell-cycle-activating phosphatase overexpressed in breast cancer, with poor prognosis, which enhances the stability of FOXO1, and thereby directly stimulates the transcription of MMP1 [145]. The least mentioned member of the FOXO family in relation to cancer FOXO6 is downregulated in breast cancer tissue compared to adjacent normal tissue, and also in breast cancer cell lines compared to the non-tumorigenic breast epithelial cell line MCF10A [146]. Downregulation of FOXO6 promotes tumor invasion, because FOXO6 transcriptionally regulates Sirt6, which suppresses the secretion of MMP9 [146].

Interestingly, the only data on aquaporins and the FOXO family were obtained on salivary gland cells where AQP5 was found to be regulated by FOXO1 [147]. However, in breast cancer the FOXO family is down-regulated, while AQP5 is up-regulated and positively correlated with its malignancy [30,54,148]. Based on these contradictory data the interaction of these molecules and their regulation in breast cancer needs to be elucidated in order to understand their role in malignancy and therapy resistance.

## 4. Conclusions

Cancer therapies, radiotherapy, chemotherapy, and immunotherapy, directly cause oxidative stress as a mechanism of action against cancer cells or indirectly as a disturbance of cellular homeostasis. Therefore, it is not surprising that factors controlling cellular oxidative stress are dysregulated in cancer cells and are often reported as factors indicating a poor outcome. We point here to three classes of proteins peroxiporins (aquaporins that channel hydrogen peroxide), NRF2, the major antioxidant transcription factor and the FOXO family of transcriptional factors along with their potential cumulative role in therapy resistance. Peroxiporins are relatively new players in cancer research and the mechanisms of their regulation as well as the interaction by which they achieve their role in the cellular process are not fully understood. Unfortunately, these three individual pathways/proteins (families) are not studied for relations between each other due to the complexity of their nature. Still, understanding their activity and possible interactions could provide new approaches in targeting breast cancer resistance. We propose here that these proteins should be investigated for their interactions, particularly with regard to therapy resistance in cancer.

## Figures and Tables

**Table 1:** Peroxiporins and breast cancer.

Studies	Peroxiporins	Models	Findings
[18]	AQP7	Mouse models Cell lines (4T1, EpH4, NMuMG) Samples of breast cancer patients	- Decreased expression of Aqp7 reduce tumor burden and metastasis in mouse models, affecting lipid metabolism, glutathione metabolism, and urea/arginine metabolism. - Converse effect of decrease in the expression of Aqp7 between normal and malignant cells: - In normal cells: promotes migration and branching while decreasing mTOR signaling; - In cancer cells: inhibits cancer progression, increases mTOR signaling, and sensitizes cancer cells to oxidative stress. - High AQP7 expression associated with unfavorable prognosis and lower overall survival in breast cancer patients.
[30]	AQP3 and AQP5	TNBC patients	- High expression of AQP3 and AQP5 observed in 60% TNBC, stronger in carcinoma tissues than in adjacent normal tissue. - AQP5 particularly pronounced at the invasive front of the tumor but decreased near necrotic areas. - In adjacent normal tissues, weak immunostaining of AQP3 and AQP5 in the periductal or intralobular stroma, with minimal expression in endothelial cells of capillaries, small veins, and peripheral nerve fibers. - Co-expression of AQP5 and AQP3 is associated with a poorer prognosis and could potentially serve as a prognostic marker for TNBC patients.
[31]	AQP1, AQP3, and AQP5	Cell lines: endocrine-sensitive (YS1.2) endocrine-resistant (pII and MDA-MB-231) normal breast epithelial cells (MCF10A)	- Cell-type-specific expression of peroxiporins: AQP1—high expression in ER-negative breast cancer cells and very low line MCF10A and non-invasive YS1.2 cells. AQP3—highly expressed in YS1.2, followed by pII and MDA-MB-231, with the lowest expression in MCF10A cells. AQP5—highly expressed in breast cancer cells compared with MCF10A. - Cell-type-specific cellular localization during the process of blebbing which promotes cell motility and invasiveness, more associated with ER-negative breast cancer cells: AQP1—primarily diffusely distributed in the cytoplasm, translocates to some extent into the blebs. AQP3—a more pronounced movement from its preferential location in the nucleus to the blebs. AQP5—remains in the nucleus.
[32]	AQP1,AQP3, and AQP5	Cell lines (MCF7 and MDA-MB-231) as sferoids	The effect of AQP1, AQP3, and AQP5 on conventional anticancer chemotherapies (cisplatin, 5-fluorouracil (5-FU), doxorubicin, and their combination alone or with the Ras inhibitor salirasib): - Overexpression of AQP3 and AQP1 sensitize MCF7spheroids to all treatments, except for 5-FU in AQP1-overexpressing spheroids (20% increase in viability). - Overexpression of AQP5—smaller spheroids with unchanged viability in MCF7, while reduced viability with no size change in MDA-MB-231. - Overexpression of AQP5—sensitize spheroids only to certain combinations of conventional chemotherapy (doxorubicin, the combination of doxorubicin, 5-FU and cisplatin, with or without salirasib) suggesting AQP5-mediated activation of the Ras pathway in MCF7 and not MDA-MB-231.
